# Hospitals’ Cybersecurity Culture during the COVID-19 Crisis

**DOI:** 10.3390/healthcare9101335

**Published:** 2021-10-07

**Authors:** Anna Georgiadou, Ariadni Michalitsi-Psarrou, Fotios Gioulekas, Evangelos Stamatiadis, Athanasios Tzikas, Konstantinos Gounaris, Georgios Doukas, Christos Ntanos, Luís Landeiro Ribeiro, Dimitris Askounis

**Affiliations:** 1 Decision Support Systems Laboratory, National Technical University of Athens, Iroon Polytechniou 9, 15780 Athens, Greece; amichal@epu.ntua.gr (A.M.-P.); gdoukas@epu.ntua.gr (G.D.); cntanos@epu.ntua.gr (C.N.); askous@epu.ntua.gr (D.A.); 2 5th Regional Health Authority of Thessaly & Sterea, Mezourlo, 41110 Larissa, Greece; fogi@dypethessaly.gr (F.G.); vstam@dypethessaly.gr (E.S.); atzi@uhl.gr (A.T.); kgounaris@ghv.gr (K.G.); 3 Projeto Desenvolvimento Manutenção Formação e Consultadoria-PDMFC, Rua Fradesso da Silveira n. 4, Piso 1 B, 1300-609 Lisbon, Portugal; luis.ribeiro@pdmfc.com

**Keywords:** cybersecurity culture, COVID-19, security assessment, phishing, health domain

## Abstract

The coronavirus pandemic led to an unprecedented crisis affecting all aspects of the concurrent reality. Its consequences vary from political and societal to technical and economic. These side effects provided fertile ground for a noticeable cyber-crime increase targeting critical infrastructures and, more specifically, the health sector; the domain suffering the most during the pandemic. This paper aims to assess the cybersecurity culture readiness of hospitals’ workforce during the COVID-19 crisis. Towards that end, a cybersecurity awareness webinar was held in December 2020 targeting Greek Healthcare Institutions. Concepts of cybersecurity policies, standards, best practices, and solutions were addressed. Its effectiveness was evaluated via a two-step procedure. Firstly, an anonymous questionnaire was distributed at the end of the webinar and voluntarily answered by attendees to assess the comprehension level of the presented cybersecurity aspects. Secondly, a post-evaluation phishing campaign was conducted approximately four months after the webinar, addressing non-medical employees. The main goal was to identify security awareness weaknesses and assist in drafting targeted assessment campaigns specifically tailored to the health domain needs. This paper analyses in detail the results of the aforementioned approaches while also outlining the lessons learned along with the future scientific routes deriving from this research.

## 1. Introduction

Coronavirus disease 2019 (COVID-19) is an infectious disease caused by severe acute respiratory syndrome coronavirus 2 (SARS-CoV-2) [1]. It was originally identified in December 2019 in Wuhan [2], from where it spread worldwide, leading to a pandemic, as denoted by the World Health Organization (WHO), in March 2020 [3]. Since then, there have been 198,778,175 confirmed cases of COVID-19, including 4,235,559 casualties [4]. As of 14 June 2021, a total of 2,310,082,345 vaccine doses have been administered, attempting to armor humans against this virus.

Even though epidemiologists argue that the health crisis is close to being over, the same does not apply to its political, societal, economic, and technical side-effects. Special circumstances created by this extraordinary crisis led to what is known as the “Great Shutdown” or “Great Lockdown” [5,6,7,8], radically altering our daily reality. Digital transformation and adaptation were forced in almost all aspects of the business world. Remote working, commonly known as “tele-working” or “working from home”, became a necessity even for sectors where it was considered prohibited up until now [9,10].

The accruing anxiety and generic crisis conditions provided a fertile ground for opportunistic criminals to act. A significant cyber-crime increase was denoted during the pandemic [11,12,13], with a noticeable preference towards the health sector [14,15,16]. Phishing, ransomware, and distributed denial-of-service attacks are only a sample of the reported cyber-crime incidents during the COVID-19 crisis [17,18,19,20,21].

Cybersecurity has been one of the emerging technological challenges of this century for the health domain [22], being among each country’s critical infrastructures. Over the last years, extensive research has been conducted aiming to identify vulnerabilities and gaps in the cyber-resilience of hospitals and healthcare facilities [23,24,25,26]. Various assessment methodologies have been applied towards pinpointing mitigation techniques and cyber-defense strategies [27,28,29,30,31,32]. Yet, scientific contribution and professional evolution failed to protect the health sector during a crisis which dictated its devotion to its main purpose of curing patients and saving lives.

Most of the security agencies, organizations and experts worldwide have issued recommendations and proposed safeguard measures to assist individuals and corporations defend against cyber-crime [11,33,34,35]. Security officers have become aware of the great cybersecurity perils they are facing. Therefore, the vast majority of them has designed and conducted a series of security awareness training programs carefully trimmed to the needs and the busy schedule of their workforce.

This paper presents the effort made by the IT and security experts of European health representative organizations during the pandemic aiming to endorse the cybersecurity awareness of healthcare employees. Towards that end, a virtual workshop was designed and held on the 16 December 2020 in Greece [36]. The effectiveness of the security awareness training program was assessed in a two-phase evaluation: a questionnaire filled directly after the workshop voluntarily by the participants and a phishing campaign held four months later.

This paper presents our research approach on evaluating the security readiness of the healthcare personnel during the COVID-19 pandemic, based on a holistic cybersecurity culture framework. Section 2 offers background information related to both the framework and the participating health domain representatives. Section 3 unfolds our methodological approach using a sequential switching between training and assessment steps. In Section 4, we analyze our two-phase security evaluation while underlying important results. Section 5 collectively summarizes our key findings, whereas, in Section 6, we outline a number of considerations and limitations regarding the proposed methodology. Finally, Section 7 concludes our research presentation by outlining areas of further research and potential future applications.

## 2. Background

### 2.1. Cybersecurity Culture Framework

Cybersecurity Culture Framework was developed in the context of the EnergyShield [37], a European Union (EU) project targeting cybersecurity in the Electrical Power and Energy System (EPES). It was officially introduced in 2020 [38], presenting an evaluation and assessment methodology of both individuals’ and organizations’ security culture readiness. It is based on a combination of **organizational** and **individual** security factors structured into **dimensions** and **domains**. Its main goal is to examine organizational security policies and procedures in conjunction with employees’ individual characteristics, behavior, attitude, and skills. Each security metric introduced by the framework is assessed using a variety of evaluation techniques, such as surveys, tests, simulations, and serious games.

The framework was later on correlated both with the hybrid MITRE ATT&CK Model for an OT Environment, consisted of a combination of the Enterprise and the ICS threat model [39] and with an enriched version of the Management and Education of the Risk of Insider Threat (MERIT) model [40], developed by the Secret Service and the Software Engineering Institute CERT Program at Carnegie Mellon University. Research related to both scientific directions focused on mapping the end-users’ socio-cultural behavior to specific cyber-threats.

During the COVID-19 crisis, the aforementioned framework was used to design a cybersecurity culture assessment campaign targeting critical infrastructures [41]. Its revealing findings [42] provided significant feedback to the participating EU organizations. Insights and recommendations towards enforcing their cybersecurity resilience were offered, further contributing to this research domain.

This scientific effort inspired SPHINX, an EU project aiming to enhance the cyber protection of the Health and Care IT Ecosystem [43], and triggered a collaboration activity with EnergyShield. The following paragraph presents how the cybersecurity culture framework assisted SPHINX security specialists in the design of a two-phase security awareness campaign targeting health sector personnel.

### 2.2. Cybersecurity Assessment

Approximately two months prior to the global outbreak of the COVID-19 crisis, a cybersecurity awareness assessment was conducted among Greek, Portuguese, and Romanian healthcare employees [44] in the context of the SPHINX EU Project. The findings on the IT workforce, doctors, nurses, auxiliary staff, laboratory personnel and administrative clerks indicated the necessity of performing targeted training and campaigns to mitigate the increasing number of phishing and fraud attacks and fortify hospital assets.

More specifically, the result analysis revealed that limited investment had been made in cybersecurity appliances procurement, software upgrades and hardware. Although an individual cybersecurity unit was not fully deployed in the surveyed organizations, all IT departments had firewalls, antivirus solutions, as well as backup mechanisms. Furthermore, it was noticed that the IT departments did not regularly keep log files of cybersecurity-related events or login actions. Cybersecurity-related key performance indicators (KPIs) were not being monitored. Notwithstanding, the IT workforce reported that penetration tests or associated training on cybersecurity concepts had not been conducted to assist them in reaching a higher level of readiness.

Additionally, a significant percentage of the non-IT staff stated that they were unaware of information security policies, albeit they could comprehend when a computer was hacked or infected and knew whom to contact. Moreover, many of them reported that they did not know what an email fraud is or how to identify it. Most importantly, the vast majority considered that organizational security policies would help improve their own work while indicating the necessity to attend sufficient cybersecurity training programs and/or general data protection regulation (GDPR) [45] seminars targeted exclusively to the operations of their healthcare institution.

Within this context, the SPHINX consortium defined and organized specific training activities and awareness webinars to increase the level of cybersecurity. To this end, apart from the dissemination of information material to the healthcare organizations with important indications and cybersecurity alerts, a webinar was explicitly designed and held to improve the cybersecurity skills of the IT employees during the COVID-19 period. The webinar took place in Greece, presenting state-of-the-art security practices, methods, tools, and standards to the healthcare environments. The cybersecurity culture framework, developed in the context of the EnergyShield project, was used to evaluate the effectiveness of the aforementioned training program, as presented in detail in the following paragraphs.

## 3. Methodology

**Figure 1:**
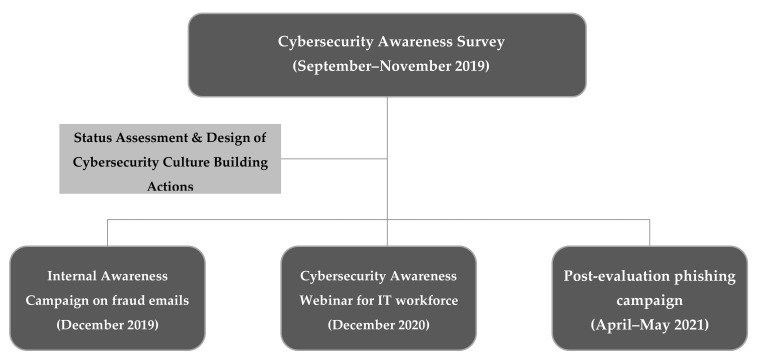
Cybersecurity Awareness Methodology.

In September 2019, a three-month cybersecurity awareness survey was held by the SPHINX consortium. After assessing 28 and 449 responses from IT and non-IT healthcare employees in Greek Healthcare Institutions [44], respectively, it was deduced that certain actions toward introducing advanced cybersecurity methods, tools, and standards were required. Therefore, an internal awareness campaign initiated by the IT departments to the rest of the healthcare staff was executed verbally or via dissemination actions. On the 16 December 2020, an IT-dedicated webinar took place [36]. The specific webinar’s effectiveness was assessed via a two-step methodology: a questionnaire filled directly after its conclusion voluntarily by the attendees and a phishing campaign held from the 26 April 2021 until the 28 May 2021. The aforementioned methodological approach is being graphically represented in Figure 1.

### 3.1. Cybersecurity Awareness Campaign

As described in the previous paragraphs, an intensive awareness campaign through the IT departments of Greek Healthcare institutions was initiated, in December 2019, focusing on actions and precautions that each healthcare employee should undertake to protect the data they handle. A variety of communication means were employed, including:

1. A certified GDPR training program provided by the Greek National Centre of Public Administration and Local Government to public servants.
2. A flyer with important cybersecurity notes and indications which was distributed to all departments and clinics. In compliance with the Directive (EU) 2016/1148 of the European Parliament and of the Council of 6 July 2016 concerning measures for a high common level of network and information systems security across the Union, the flyer informed its readers that healthcare organizations have to comply with certain cybersecurity rules regarding their network and information systems. Consequently, the healthcare workforce was advised to:

- Change the access passwords frequently without disclosing them;
- Always keep backup of critical data (if possible);
- Avoid opening emails and following links from unknown senders without first checking the sender’s emails;
- Never allow unauthorized third parties to use the organizations’ workstations;
- Always lock their screens prior to leaving the office;
- Avoid plugging in a USB stick on the PCs without the approval of the IT department.

### 3.2. Cybersecurity Awareness Webinar

In December 2020, a cybersecurity awareness webinar was specifically designed trimmed to the needs of the Greek IT health domain departments. The webinar was made publicly available (upon registration) to every EU healthcare IT employee interested in participating. Instructors from the European Union Agency for Cybersecurity (ENISA), academic institutions and cybersecurity industry representatives from the SPHINX consortium were involved. The webinar presented aspects from ISO 27001 [46] as a path to the directive on security of network and information systems (NIS directive) compliance [47]. Moreover, it highlighted the key points to cybersecurity risk assessment in hospitals along with procurement guidelines for healthcare cybersecurity. Furthermore, various practical methods and techniques were presented to assist IT employees in their daily activities to control cybersecurity while topics in the state-of-the-art firewalls, antivirus configurations, backup mechanisms as part of the network topologies were covered.

After the webinar’s conclusion, the participants were requested to respond to a questionnaire, voluntarily and anonymously, in order to measure the comprehension level of the concepts presented. The questionnaire included questions on demographics, information security and policies, network security and data management (Appendix A). From a total of 113 attendees from various EU countries and institutions, 62 were employed in Greek Hospitals’ IT departments (approximately 30% of the total permanent IT workforce of Greek healthcare organizations in the public sector [48]), and 30 of them answered the optional questionnaire.

### 3.3. Phishing Awareness Campaign

Based on the 2020 HIMSS Healthcare Cybersecurity Survey, security incidents continue to plague healthcare organizations of all types and sizes, with phishing being the most common of all [49]. Phishing is a social engineering tactic that is used to persuade individuals to provide sensitive information. Malicious actors employ phishing techniques for a variety of reasons, including identity theft, access to proprietary information, transmission of malicious software to include ransomware, unauthorized remote access, and initiation of unauthorized financial transactions [50]. The most common form of phishing is the **phishing email** which usually attempts to appeal to a recipient’s fear, duty, obligation, curiosity, or greed [51].

In late January 2020, Coronavirus-themed Emotet spam campaigns were reported, primarily targeting Japanese entities [52,53]. From January to April 2020, Interpol detected about 907,000 spam messages tied to COVID-19 [54]. During April 2020, Google reportedly blocked more than 18 million malware and phishing emails related to COVID-19 and in addition to more than 240 million COVID-related daily spam messages [55].

Consequently, and as a final methodological step, a cybersecurity culture assessment campaign was sketched aiming to post-evaluate the health domain’s workforce familiarity with phishing email techniques in specific. Recent research shows a statistically significant positive correlation between workload and the probability of health care staff opening a phishing email [28]. Therefore, we decided to create a phishing quiz, instead of a simple questionnaire, including several different phishing emails. Its duration needed to be short to ensure the commitment and concentration of the participants given their extremely heavy workload and resulting fatigue.

A phishing simulation exercise–where the participants would receive a phishing email without prior knowledge, containing a link they should not click on-could have been a more realistic approach towards evaluating the actual workforce behavior given the concurrent circumstances. Yet, such an approach was rejected by the collaborating IT experts after extensive discussions. One of the main reasons was that such an evaluation exercise would suggest a significant effort in altering the configuration of the existing security solutions in place to allow those “phishing” emails to reach their targeted participants. Moreover, participants needed to be informed and consent to become part of this security evaluation campaign. Due to the psychologically and emotionally demanding period of the COVID-19 pandemic, it was agreed that most people would willingly take a short quiz initiated on-demand and in their time of choice rather than accept to be evaluated via a simulation test performed over a specific period of time. The latter would significantly increase the evaluation stress and, therefore, decrease the participation rate.

Phishing emails that were either blocked by the deployed antispam solutions or communicated to the IT departments by the healthcare recipients and processed accordingly based on the applied security protocols have been gathered by SPHINX security experts and collaboratively examined for similarities and differences. After a number of evaluation sessions, they concluded with the five emails presented in Table 1. 

The specific survey targeted hospitals’ workforce during the COVID-19 crisis. A significant percentage of the IT staff, technicians and administrative clerks exercised teleworking due to the COVID-19 restrictions opposite to the medical, nursing and laboratory personnel that had no such alternative. Therefore, our main goal was to evaluate the familiarity of non-medical personnel with phishing email techniques and assess their readiness while in teleworking conditions and following previous cybersecurity training and familiarity campaigns (Table 2).

IT: employees working in the information technology department; technicians: employees working in the electro-mechanical and biomedical departments; clerks: employees working in the accounting, finance, and procurement departments.

A special invitation email was sent to the selected participants providing a connection link and appropriate authentication credentials. Each participant was able to complete only once the phishing quiz, with no time limitations, and had to provide an answer to each one of the emails included in the campaign. Both the invitation email and the phishing quiz were localized, ensuring proximity, and lifting language barriers usually introduced to such evaluations.

**Figure 2:**
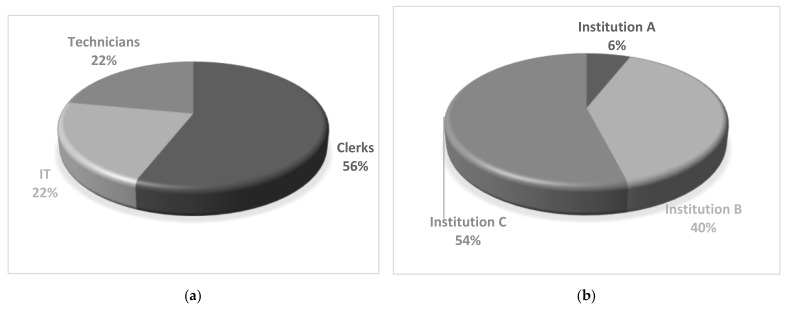
Campaign General Participation Information: (**a**) Expertise, (**b**) Healthcare Institution.

The campaign was available for participation for almost a month, starting from 26 April 2021 and ending on 28 May 2021. During that period, all 50 invited participants completed the phishing quiz anonymously, thus, achieving 100% participation rate. Participation rate varied based on the hospitals’ patient capacity concluding to a 54% from Institution A, 40% from Institution B and 6% from Institution C. More specifically, 56% of the participants were clerks, 22% were IT professionals, and 22% were technicians (as presented in Figure 2).

## 4. Detailed Assessment Results

### 4.1. Cybersecurity Awareness Webinar Results Analysis

Immediately after the conclusion of the cybersecurity awareness webinar, participants were asked to complete a questionnaire (presented in Appendix A) voluntarily and anonymously. Based on its results Table 3, 56.7% of the participants were aged between 40–49 years old, while 43.3% were female. Moreover, 56.7% held an MSc, while 80.0% had more than ten years of working experience in the field of healthcare IT. Around 70.0% were employed in hospitals, and 33.3% held managerial positions, while 36.7% worked for healthcare institutions that employ more than 1201 healthcare professionals.

**Figure 3:**
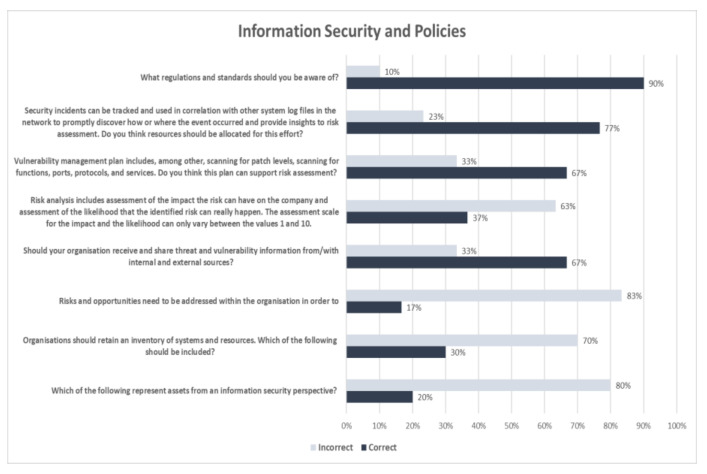
Evaluation of the Information Security and Policy Comprehension.

Figure 3 presents the questionnaire results associated with information security and policies. More specifically, 90% responded correctly that Health Insurance Portability and Accountability Act (HIPAA) [56] and ISO/IEC 27799 (Health informatics—Information security management in health using ISO/IEC 27002) [57] standards are those they should be aware of, while the rest of the participants (10%) answered incorrectly that COBIT and ITIL or PCI/DSS and SOX should be taken into consideration. Furthermore, in the question related to the resources’ allocation towards the discovery of cybersecurity events, 77% replied correctly that resources should be exclusively allocated to this task, while 23% considered that it would be better to allocate these resources elsewhere or that resources should be allocated based on the availability of an IT team. A total of 67% of the responders correctly stated that a vulnerability management plan that includes, among others, scanning for patch levels, functions, ports, protocols, and services could support risk assessment in comparison to 33% that replied negatively or were unaware. Only 37% replied correctly that the assessment scale for the impact and the likelihood could not only vary between the values one and ten, while 63% replied either positively or ignorant.

Around 67% answered correctly that it was necessary for their organization to receive and share threat and vulnerability information from/with internal and external sources. Regarding the multiple answers question about the necessity to address risk and opportunities within their organization, only 17% responded that it was required to both prevent and reduce undesired effects and achieve continual improvement. The rest—83%, answered either partially or in combination with other alternatives. Only 30% replied correctly that every organization asset should be encompassed in the inventory of systems and resources, while 70% replied partially correctly to the question. Finally, 20% replied correctly that people, software, and paper-based information represented assets from an information security perspective. The rest—80%, responded only partially correctly or considered that unauthorized modification or low awareness of information security could be assets too.

**Figure 4:**
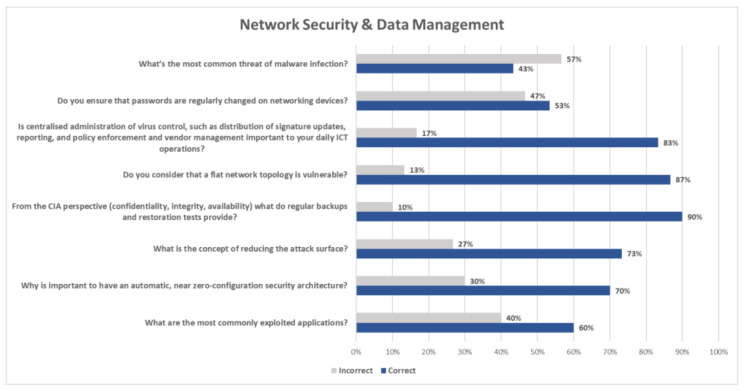
Evaluation of the Comprehension of Network Security and Data Management.

Figure 4 collectively presents answers to questions associated with network security and data management. More specifically, this part of the questionnaire revealed that 53% of the participants prefer a standard password expiration policy at regular intervals, while 47% stated they prefer to change the default passwords and, thereafter, not asking end-users to change their passwords. A total of 83% of the responders considered that a centralized administration of virus control, such as distribution of signature updates, reporting, policy enforcement and vendor management, was important to their daily IT operations because it helped them do their work faster and real-time monitor their assets. On the other hand, 17% replied that they had manually installed antivirus software to their assets and consequently did not consider this an important security policy. The vast majority (87%) recognized a flat network topology as a vulnerable architecture. Furthermore, from a CIA perspective (confidentiality, integrity, availability), 90% replied that regular backups and restoration tests ensured availability and reduction of the recovery time in restoring a system to operational mode. On the other hand, 10% stated ignorant or that only backups were important for availability, reducing the risk of losing data. Further, 73% responded correctly that the concept of reducing the attack surface involved segmentation of network zones, blocking of activities associated with vulnerabilities and combating malicious code. In addition, 27% replied partially correct by selecting only one from the aforementioned actions.

Furthermore, 70% answered that it was important to have an automatic, near zero-configuration security architecture because it reduced manual labor and human error, while 30% added incorrectly that it would also be cheaper and easier to implement. In addition, 60% replied correctly that the most commonly exploited application is the Office Suite, while the rest 40% reported either browsers, operating systems, JAVA or PDF files. Moreover, 43% stated correctly that Trojans were the most common threat of malware infection while the rest 57% answered adware, viruses, or potentially unwanted programs. When questioned if intrusion detection and intrusion prevention software was considered as one of the important components in edge security, 63% replied positively having active subscription while the rest 37% responded positively too without having an active subscription, considering though to procure it in the future.

### 4.2. Phishing Awareness Campaign Results Analysis

**Figure 5:**
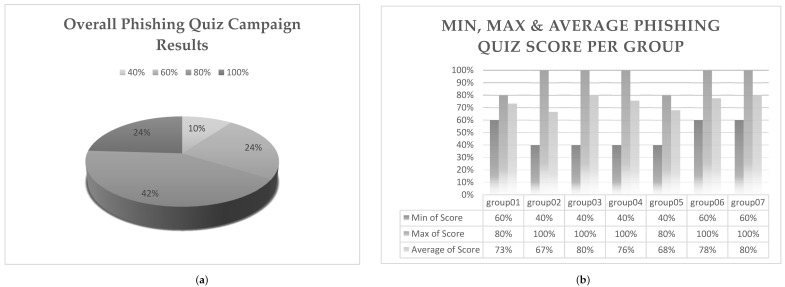

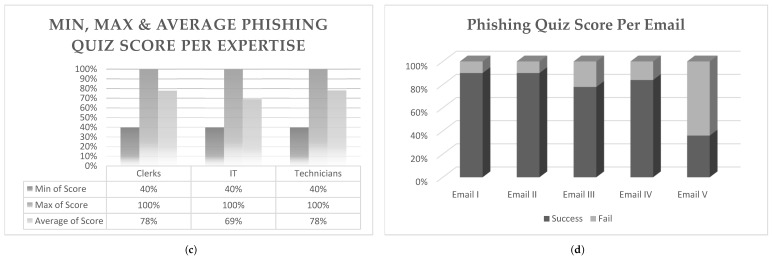
Campaign generic assessment results: (**a**) overall, (**b**) per group, (**c**) per expertise and (**d**) per email.

Based on the phishing quiz results, as presented in Figure 5, 1 out of 4 participants was able to distinguish a legit from a phishing email with a 100% success score. Only 10% of them did not manage to obtain a passing score since they only identified two out of five emails. Although such a score would be considered quite satisfying in many cases, the same does not apply to the cybersecurity reality where an organization is as strong as its weakest link.

When examining the overall campaign’s results from a group perspective, as depicted in Figure 5b, we notice that five out of seven groups managed to achieve a score higher than 70%. Probably, a disturbing observation, though, is that IT personnel appears to bear the lowest average in comparison with the rest of the groups, meaning the clerks and the technicians (Figure 5c). Due to the close correlation of the Information Technology and Information Security domains, a better cybersecurity awareness and phishing techniques’ familiarity was expected of the IT experts.

**Figure 6:**
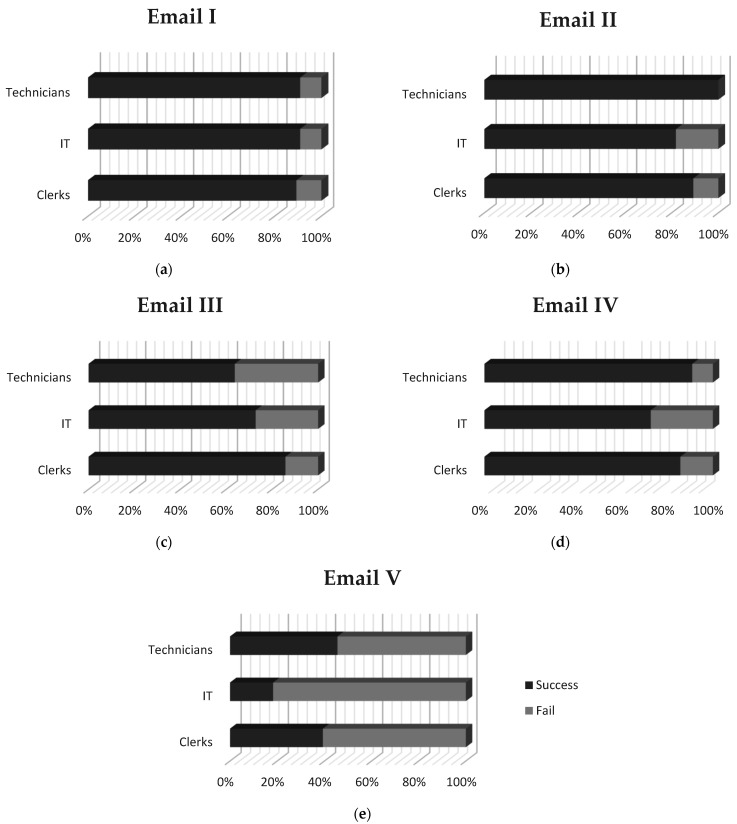
Campaign assessment results per expertise for: (**a**) email I, (**b**) email II, (**c**) email III, (**d**) email IV and (**e**) email V, of the phishing quiz.

Narrowing down to achievement scores per email, Emails I and II appear to have better phishing identification scores (higher than 80% by all participating groups), as presented in Figure 5d and Figure 6. Interestingly, these two emails bear no similarities. The first one, as presented in Table 1, is related to a bank institution, containing an easily recognizable logo and seeking account verification by clicking on a hyperlinked text where a suspicious redirection is being hidden. The second one is quite long, containing only text and attempting to convince, using slang language, its recipients to pay an amount of ransom in Bitcoin in order not to reveal personal videos recorded via their hacked workstation cameras. Phishing techniques used in these two cases are quite different and usually aim at different target audiences. Email I have an appeal on a recipient’s sense of duty and punctuality, whereas Email II on fear and uncertainty. Yet, hospital employees participating in this evaluation campaign managed in their majority to recognize both of them as not legit.

One would expect that Email III would present similar results with Email I since, as presented in Table 1, they look alike. Email III is also related to a bank institution, containing its logo, seeking account verification by providing a hyperlink that is not hidden but instead is fully visible to its readers. Therefore, better results were anticipated since less effort was needed to locate the misleading redirection. Since it was the third entry in the phishing quiz, boredom and carelessness could be taking the lead from caution and reservedness explaining the degrading scores. However, such a conclusion would not agree with the results noticed for Email IV, as depicted in Figure 5d and Figure 6, where scores are improved.

Last but not least, we notice that the majority of the participants (64%) failed to identify the only legit email included in the phishing quiz. The specific email was short (no more than 38 words), containing no images or logos, no special font formatting or email structures (e.g., tables). The word “here” was used to provide a hidden hyperlink (could be previewed when the user hovered over the word with the mouse) which could be easily acknowledged that it redirects to the legit Ministry of Internal Affairs website. Even though the specific result could be attributed to the increased cautiousness of the users due to the special circumstances of the crisis and the nature of the assessment, it remains quite disturbing. Legit emails might be forwarded for security analysis, rejected, or even deleted without communicating their context to their recipients due to them being erroneously identified as phishing attempts.

## 5. Key Findings

The analysis of the webinar’s questionnaire showed that the IT departments comprehended sufficiently concepts such as standards’ application to their policies and the incorporation of iterative risk assessment of their assets among their operations. Additionally, they exhibited high familiarity with the various network topologies and advanced cybersecurity tools. However, more emphasis should be given to focused training programs targeting risk assessment and data asset identification. It is deduced that healthcare IT employees are highly aware of cybersecurity concepts and how to protect their network and information systems.

Summing up the results of the targeted post-assessment campaign on phishing, the most apparent and at the same time unexpected observation is that the lowest average score is attributed to the IT professionals. They were expected to be the most qualified of the respondents and the ones apt to guide and advise the hospitals’ personnel on their actions with respect to suspicious emails. However, these results came after a series of Emotet spam campaigns that affected their hospitals. These events can have reasonably sensitized their awareness and hardened their judgment. Indeed, the lowest score emerges for Email V where only 18% of the IT personnel identified successfully that that was a legit email (Figure 6). Although the above reasoning could adequately justify this result, it cannot be considered an explanation where no action is required. Behavioral awareness in cybersecurity calls for the right decisions where legit emails will reach their recipients and enjoy appropriate handling, while phishing emails will be immediately detected and rejected. Therefore, the results suggest that there is still room for dedicated training programs that should first—but not exclusively—target the hospitals’ IT departments for them to be able to offer a robust first security layer and provide the right advice when requested. Besides, the great success of phishing emails in deceiving can be attributed to the fact that phishers become smarter [58]. Therefore, even the tech-savvy people can be deceived, while regular training can certainly shield an organization, as previous works suggest [59,60].

Another observation is that there is no notable difference among the three groups of IT personnel, technicians, and clerks, as indicated by both their average scores and the individual analysis, which would constitute the one better prepared than the others. We see two explanations that can be given to that. Firstly, in general, people tend to have difficulty relating to such a theoretical problem, which they believe will not happen to them [61]. Therefore, when receiving a new email, they do not invest the time and effort to question its intentions. Secondly, more tech-savvy people tend to be overconfident in their ability over others to identify fraud and mal-intent, which usually turns to be a naive perception [61].

**Figure 7:**
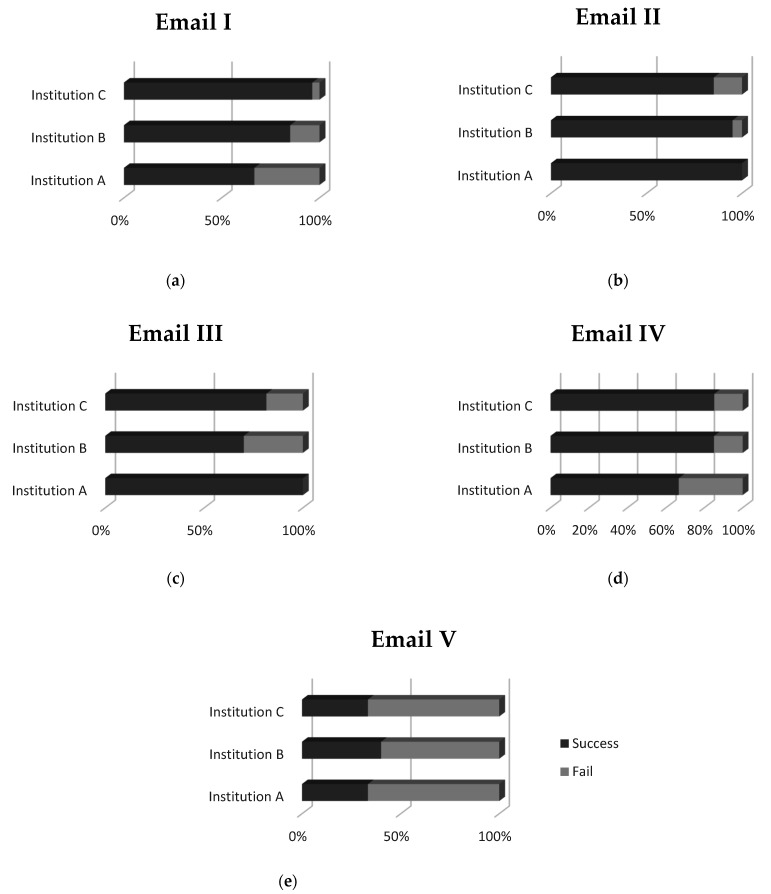
Campaign assessment results per hospital for: (**a**) email I, (**b**) email II, (**c**) email III, (**d**) email IV and (**e**) email V, of the phishing quiz.

Finally, the analysis results yielded no noteworthy differences among the three Greek healthcare institutions participating in the analysis. As depicted in Figure 7, the encouraging finding is that the lowest scores appear for all three hospitals for Email V, the only legit email of the phishing quiz. However, this finding should not remain unaddressed for the reasons explained previously. In general, advancing phishing email filters [62] in a way that would ensure that only the bare minimum of phishing emails and only rarely will remain undetected and surpass the filter would well safeguard the hospitals and take the weight of increased awareness off the employees’ shoulders. Experience has shown, though, that a perfect phishing email filtering mechanism could not exist, and the recipients’ cybersecurity awareness is the key to phishers’ failure.

## 6. Considerations and Limitations

The security awareness webinar and the post-evaluation phishing campaign were conducted during the COVID-19 crisis. Cyber-attacks against critical infrastructures were on the rise, while, on parallel, the health sector necessitated advanced cybersecurity protection mechanisms and enhanced security culture as this is introduced by an organization’s human capital. In this context, we aimed at informing the hospitals’ personnel regarding concurrent cybersecurity risks and mitigation strategies against them. We then evaluated their cybersecurity resilience using both a simplified questionnaire and a phishing campaign. The prioritization of the phishing quiz campaign against the other alternatives provided by the Cybersecurity Culture Framework presented in Section 2.1 was set by the IT and security experts of the participating hospitals, giving their alarming frequency. A phishing simulation exercise, which could also serve the same purpose, was rejected, after careful consideration, due to the extra effort required by the IT and security personnel to properly configure and by-pass the anti-spam solutions in place. Concerns related to ensuring a high participation rate without further disrupting or stressing participants were also in favor of the phishing quiz approach.

Due to COVID-19 and the profoundly heavy schedule of the medical staff, we decided not to engage them at this stage, which, of course, restricted the extent of our analysis and the application scope and generalizability of its findings. Our next steps involve engaging a fair sample of the medical staff of these three hospitals in the campaign when conditions permit it. This will allow a complete understanding of the hospitals’ readiness concerning phishing attacks since staff from all key roles of the hospitals’ operation will have been engaged.

Another limitation is the fact that the campaign was restricted in Greece; thus, not making possible the comparison of the cybersecurity culture in the health sector among countries in the EU or even globally. Furthermore, the selection of five emails (four of them not being legitimate) for the phishing quiz that the participants had to take might be considered small and not adequate for assessing one person and his security behavior. However, the engagement of a satisfactory number of the hospitals’ staff in the campaign and their focus during the quiz’s completion were the top priorities, susceptible to non-satisfaction if an enlarged, more complex quiz had been given. In parallel, these five emails were proven enough to highlight potential gaps and weaknesses in Greek hospitals’ security culture and pinpoint new training routes.

## 7. Conclusions and Future Work

The current manuscript aimed to explore cybersecurity culture of the hospitals’ personnel during the COVID-19 pandemic. A questionnaire examined participants’ knowledge and familiarity with information security concepts, policies, procedures, and practices, while a phishing campaign focused on their attitude and behavior towards phishing techniques; probably the most disturbing security issue faced during the COVID-19 crisis. The assessment’s design was based on a robust methodology, which is part of a broader context, the Cybersecurity Culture Framework presented in Section 2.1. Three Greek hospitals participated in the evaluation campaign with staff members belonging to one of the following three groups: IT professionals, technicians, and clerks.

In that view and given the previously identified considerations concerning the current work, our next steps involve extending the analysis in three levels: (a) participants’ involvement and role in the hospital, (b) the examined security dimensions of the cybersecurity culture framework, and (c) the geographical coverage. Two new cybersecurity culture assessment campaigns are now planned, aiming after the first and second levels, respectively. In particular, the first campaign aims to continue the current phishing campaign involving more staff members, focusing on the medical staff, to allow a full overview of the participating hospitals’ readiness concerning phishing attacks. The second campaign aims to involve and examine more security dimensions of the cybersecurity culture framework through an effective combination of questionnaires, tests, simulations, and serious games targeted to the background and needs of the health sector. This campaign will focus on selected personnel with key roles with respect to security in the participating hospitals. The extension of these campaigns to more countries will follow the completion of the objectives mentioned above.

## Figures and Tables

**Table 1:** Emails Used in The Evaluation Campaign.

ID	Description	Phishing	Legit
Email I	X Bank asking recipients to protect their accounts by following a specific hyperlink.	✓	
Email II	Unknown sender blackmailing recipients asking for ransom in Bitcoin in order not to reveal personal videos recorded via their hacked workstation cameras.	✓	
Email III	Y Bank asking recipients to protect their accounts by following a specific hyperlink.	✓	
Email IV	An email supposedly sent by the IT department asking for account verification to avoid inactivation.	✓	
Email V	An email related to the Ministry of Internal Affairs deriving from the repository of public expenditures.		✓

**Table 2:** Groups of Users Participating in The Evaluation Campaign.

	IT	Technicians	Clerks
**Institution A**	group01 (user01–user03)		
**Institution B**	group02 (user04–user06)	group03 (user07–user09)	group04 (user10–user23)
**Institution C**	group05 (user24–user28)	group06 (user29–user36)	group07 (user37–user50)

**Table 3:** Demographics of Workshop Participants That Answered the Questionnaire.

Category	Participants
Total	*n* = 30 (100%)
**Gender**	
Male	17 (56.7%)
Female	13 (43.3%)
**Age**	
20–29	2 (6.7%)
30–39	6 (20.0%)
40–49	17 (56.7%)
50–59	5 (16.7%)
**Education**	
Secondary Education	2 (6.7%)
Bachelor’s degree	7(23.3%)
MSc	17 (56.7%)
PhD	4 (13.3%)
**Years of Experience**	
0–5	5 (16.7%)
6-10	1 (3.3%)
> 10	24 (80.0 %)
**Position**	
ICT staff	12 (40.0%)
ICT manager	10 (33.3%)
ICT director	3 (10.0%)
Other	5 (16.7%)
**Organization**	
Hospital	21 (70.0%)
Health Authority	3 (10.0%)
Other	6 (20.0%)
**Number of Employees in your Organization**	
<100	4 (13.3%)
100–300	2 (6.7%)
301–600	7 (23.3%)
601–1000	3 (10.0%)
1001–1200	3 (10.0%)
>1201	11 (36.7%)

ICT: Internet and Communication Technologies.

## Data Availability

The data presented in this study are available on request from the corresponding author.

## Appendix A

***General Characteristics***-***Demographics***

1. Country

  *(Free Text)*

2. Age

  20–29 ☐ 30–39 ☐ 40–49 ☐ 50–59 ☐ 60 + ☐

3. Gender

  Male ☐ Female ☐

4. Education

Secondary Education	☐
Vocational training Institution	☐
Bachelor’s Degree	☐
MSc	☐
PhD	☐

5. Position

ICT director	☐
ICT manager	☐
ICT personnel	☐
Other	☐

6. Years of experience

6–10	☐
more than 10	☐

7. Organization

Hospital	☐
Clinic	☐
Health Authority	☐
National	☐
Regional	☐
Local	☐
Other	☐

8. Number of Employees in your Organization

Employees <100	☐
Employees 100–300	☐
Employees 301–600	☐
Employees 601–1000	☐
Employees 1001–1200	☐
Employees > 1201	☐

***Information Security and Policies***

9. Which of the following represent assets from an information security perspective?

People	☐
Unauthorized modification	☐
Software	☐
Low awareness of information security	☐
Paper-based information	☐

10. Organizations should retain an inventory of systems and resources. Which of the following should be included?

Every device, including computers, tablets, routers, printers, servers, and phones, on the network	☐
Only important network resources	☐
Information regarding connection types and data types	☐
Only network resources for which there is available information	☐
Information regarding the departments with access to systems, and their vendors	☐

11. Risks and opportunities need to be addressed within the organization in order to:

Demonstrate IT team readiness	☐
Prevent or reduce undesired effects	☐
Achieve continual improvement	☐

12. Ensure all employees are aware of the risks and opportunities ☐ Should your organization receive and share threat and vulnerability information from/with internal and external sources?

Yes	☐
No	☐
Don’t Know	☐

13. Risk analysis includes assessment of the impact the risk can have on the company and assessment of the likelihood that the identified risk can really happen. The assessment scale for the impact and the likelihood can only vary between the values 1 and 10.

Yes	☐
No	☐
Don’t Know	☐

14. Vulnerability management plan includes, among other, scanning for patch levels, scanning for functions, ports, protocols, and services. Do you think this plan can support risk assessment?

Yes	☐
No	☐
Don’t Know	☐

15. Security incidents can be tracked and used in correlation with other system log files in the network to promptly discover how or where the event occurred and provide insights to risk assessment. Do you think resources should be allocated for this effort?

Yes, resources should be allocated exclusively for this purpose	☐
Yes, resources should be allocated based on the availability of ICT team	☐
No, it is better to allocate these resources elsewhere	☐
Don’t Know	☐

16. What regulations and standards should you be aware of?

HIPAA and ISO/IEC 27799	☐
PCI/DSS and SOX	☐
COBIT and ITIL	☐

***Network Security & Data Management***

17. What’s the most common threat of malware infection (select only one from the following)?

Trojans	☐
Potentially Unwanted Programs	☐
Viruses	☐
Adware	☐
Worms	☐

18. Do you consider Intrusion Detection / Intrusion Prevention Software as one of the important components in the edge security?–

Yes, and our department uses it with active subscription	☐
Yes, but our subscription has expired	☐
Yes, and we consider purchasing in the future	☐
No, it is not important	☐
Don’t Know	☐

19. What are the most commonly exploited applications (select only one from the following)?

Operating systems, Win/Linux/MacOS	☐
Mobile Operating Systems, Android/IOS	☐
Browsers	☐
Office Suite	☐
Java	☐
Flash	☐
PDF	☐

20. From the CIA perspective (confidentiality, integrity, availability) what do regular backups and restoration tests provide?

Ensure availability and reduce the recovery time to restore a system back to operational mode	☐
Only Backups are important for availability, since they reduce the risk of losing all your data	☐
Don’t Know	☐

21. Do you consider that a flat network topology is vulnerable?

No, and it is used to easily administrate the network	☐
Yes, because once a node is breached it has access to every other one on the same network	☐
Don’t Know	☐

22. Is centralized administration of virus control, such as distribution of signature updates, reporting, and policy enforcement and vendor management important to your daily ICT operations?

No, as soon as we have manually installed antivirus software to our assets	☐
Yes, because it helps to do our work faster and real-time monitor our assets	☐
Don’t Know	☐

23. Do you ensure that passwords are regularly changed on networking devices?

No, as soon as we have changed the default passwords	☐
Yes, and we do this twice per year	☐
Don’t Know	☐

24. What is the concept of reducing the attack surface?

Segment network zones	☐
Block activities associated with vulnerabilities and combat malicious code	☐
All of the above	☐

25. Why is important to have an automatic, near zero-configuration security architecture

It reduces manual labor and human error	☐
It will be cheaper and easier to implement	☐
All of the above	☐
